# Discovery and Chromosomal Location a Highly Effective Oat Crown Rust Resistance Gene *Pc50-5*

**DOI:** 10.3390/ijms222011183

**Published:** 2021-10-17

**Authors:** Joanna Toporowska, Sylwia Sowa, Andrzej Kilian, Aneta Koroluk, Edyta Paczos-Grzęda

**Affiliations:** 1 Institute of Plant Genetics, Breeding and Biotechnology, University of Life Sciences in Lublin, Akademicka 13 St., 20-950 Lublin, Poland; joanna.toporowska@up.lublin.pl (J.T.); aneta.koroluk@up.lublin.pl (A.K.); 2 Diversity Arrays Technology Pty Ltd., University of Canberra, Canberra, Kirinari St., Bruce, ACT 2617, Australia; zej@diversityarrays.com

**Keywords:** *Puccinia coronata* f. sp. *avenae*, DArT*seq*, marker assisted breeding, *Avena sativa* L. genome, molecular mapping

## Abstract

Crown rust, caused by *Puccinia coronata* f. sp. *avenae*, is one of the most destructive fungal diseases of oat worldwide. Growing disease-resistant oat cultivars is the preferred method of preventing the spread of rust and potential epidemics. The object of the study was *Pc50-5*, a race-specific seedling crown rust resistant gene, highly effective at all growth stages, selected from the differential line Pc50 (*Avena sterilis* L. CW 486-1 × Pendek). A comparison of crown rust reaction as well as an allelism test showed the distinctiveness of *Pc50-5*, whereas the proportions of phenotypes in segregating populations derived from a cross with two crown rust-susceptible Polish oat cultivars, Kasztan × Pc50-5 and Bingo × Pc50-5, confirmed monogenic inheritance of the gene, indicating its usefulness in oat breeding programs. Effective gene introgression depends on reliable gene identification in the early stages of plant development; thus, the aim of the study was to develop molecular markers that are tightly linked to *Pc50-5*. Segregating populations of Kasztan × Pc50-5 were genotyped using DArT*seq* technology based on next-generation Illumina short-read sequencing. Markers associated with *Pc50-5* were located on chromosome 6A of the current version of the oat reference genome (*Avena sativa* OT3098 v2, PepsiCo) in the region between 434,234,214 and 440,149,046 bp and subsequently converted to PCR-based SCAR (sequence-characterized amplified region) markers. Furthermore, 5426978_SCAR and 24031809_SCAR co-segregated with the *Pc50-5* resistance allele and were mapped to the partial linkage group at 0.6 and 4.0 cM, respectively. The co-dominant 58163643_SCAR marker was the best diagnostic and it was located closest to *Pc50-5* at 0.1 cM. The newly discovered, very strong monogenic crown rust resistance may be useful for oat improvement. DArT*seq* sequences converted into specific PCR markers will be a valuable tool for marker-assisted selection in breeding programs.

## 1. Introduction

Oat (*Avena sativa* L.) is an important cereal crop used in the food industry and for animal feed and fodder [1]. Globally, it ranks seventh in grain production, reaching approximately 23 million tons, with Russia, Canada, Australia and Poland being the largest oat producing countries [2]. Oat leaf diseases are responsible for significant decreases in yield quantity and quality in all areas of oat cultivation [3]. Crown rust, caused by *Puccinia coronata* f. sp. *avenae*, is one of the most devastating fungal diseases of oat in the world [4]. The application of fungicides in oat cultivation may be economically unjustified and is potentially harmful to the environment [5,6]. Thus, growing disease-resistant oat cultivars is the most effective strategy for controlling crown rust. Therefore, it is important to develop genetically resistant genotypes.

Major resistance genes, such as *Pc38*, *Pc39*, *Pc48*, *Pc62* and *Pc68* were introduced into breeding programs in 1980 [6,7] whereas *Pc94* [8] and *Pc91* [9] were introduced in 2004 and 2005, respectively. However, the effectiveness of monogenic resistance is usually short-lived and remains effective for 3–7 years [10]. Pathogens overcome resistance genes in the plant as a result of the spread of a host plant carrying a single major resistance gene over a wide geographic region and due to changes in virulence in the pathogen population. The loss of effectiveness of the major resistance gene is the result of rapid pathogen adaptation [11]. Despite overcoming the major resistance gene, the identification of new alternative genetic sources of resistance and introducing such genes is much easier and faster than introducing durable plant resistance. Therefore, the continuous search for new sources of monogenic crown rust resistance may be useful for oat improvement. Given the economic and environmental advantages of cereal host genetic resistance over fungicide application, such efforts should be a priority [12]. The availability and utilization of diverse sources of effective resistance is crucial for successful breeding programs [12,13]. Moreover, advances in next-generation sequencing and molecular marker development have simplified the process of marker-assisted selection.

Wild populations of *Avena sterilis* L. have been widely used as donors of crown rust race-specific resistance genes [14,15,16,17]. Nearly half of the above 100 *Pc* genes originated from this species. These include *Pc35*, *Pc36*, *Pc38*-*Pc77* [14], *Pc97* [6], *Pc98* [18], *Pc101*, *Pc103* and *Pc104* [19,20]. Some of *A. sterilis* accessions were the source of more than one *Pc* gene, e.g., *A. sterilis* F-83 (*Pc40*-*Pc43),* PI 287211 (*Pc60* and *Pc61*) or CAV 4248 (*Pc64*-*Pc66*) [21,22].

The differential line Pc50 belongs to the set of nearly isogenic lines of the *A. sativa* cultivar Pendek, into which crown rust resistance genes were introgressed from *A. sterilis* CW 486-1 by backcrossing [23]. Šebesta and Harder [24] used Polish *P. coronata* isolates and reselected two lines from Pc50 differential: Pc50-2 and Pc50-4, each carrying a distinct major gene [25,26]. In our study, we also found segregation of some *P. coronata* races originated from Poland within the Pc50 line derived from the Canadian Pc differential set, suggesting the presence of another previously unidentified resistance gene, designated here as *Pc50-5*.

The objectives of the present study were to: (1) confirm the distinctiveness of *Pc50-5*, (2) determine monogenic inheritance of *Pc50-5*, (3) localize *Pc50-5* in the oat genome v.2 using DArT*seq*-derived SNPs and (4) develop specific *Pc50-*5-linked PCR markers useful for marker-assisted selection (MAS).

## 2. Results

### 2.1. Crown Rust Reaction Comparison and Segregation Analysis

**Figure 1:**
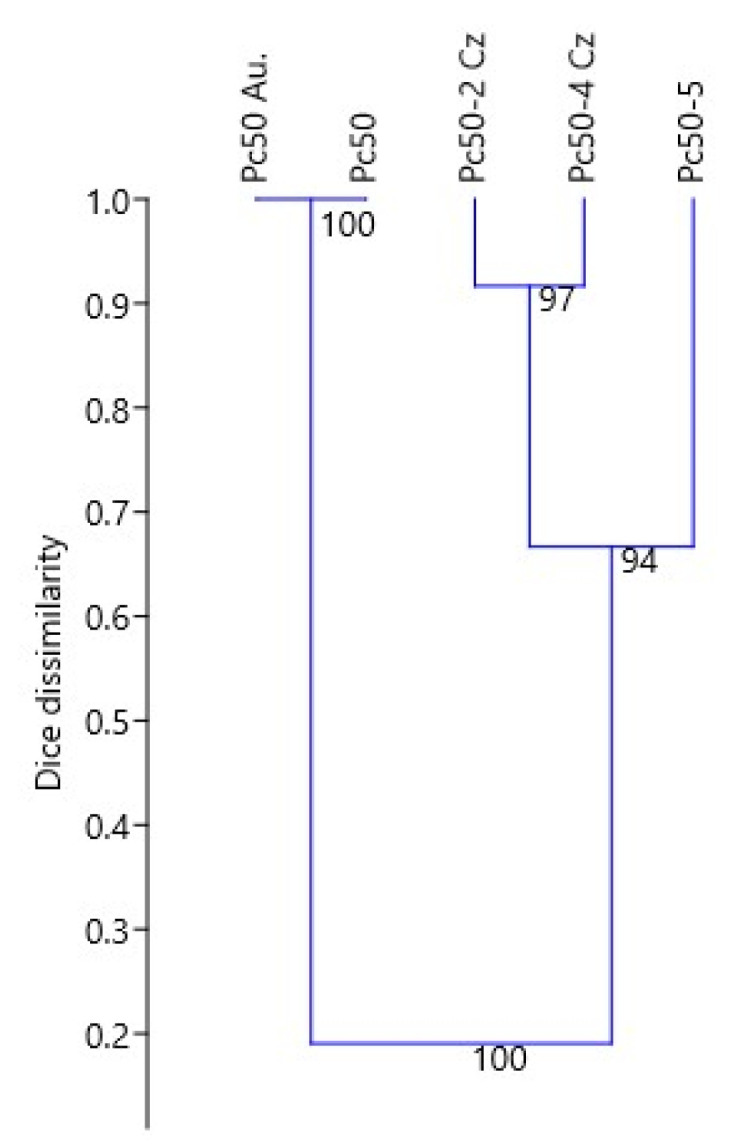
UPGMA dendrogram of oat isolines derived from Pendek × CW 486-l crossing based on the similarity of infection obtained by 14 *P. coronata* races inoculation.

The isoline Pc50 (Pendek × *A. sterilis* CW 486-l) obtained from the Cereal Research Centre AAFC, Winnipeg, Canada, was used in our previous crown rust study conducted in 2013–2019 in Poland [20]. Segregation of resistant and susceptible plants with respect to some *P. coronata* races was found within this isoline, and the subline Pc50-5 was reselected. The responses of Pc50, Pc50Au, Pc50-2, Pc50-4 and Pc50-5 to 14 *P. coronata* race inoculation were compared in the host-pathogen test to prove the distinctiveness of the newly discovered isoline Pc50-5 from the rest of the lines derived from the progeny of the seemingly homogeneous Pendek × *A. sterilis* CW 486-l hybrid. Infection severity rates were recorded, converted to binary values (Table 1) and displayed in a dissimilarity dendrogram developed based on the Dice coefficient indices (Figure 1). Hierarchical analysis identified two distinct clusters: the first consisting of the original Pc50 line from Canada and Pc50Au of Australian origin, as both lines presented identical reactions to *P. coronata* inoculation, and the second cluster comprising the strongly similar Pc50-2 and Pc50-4 lines along with the newly discovered Pc50-5 with a dissimilarity coefficient of approximately 0.65.

Two *P. coronata* races (I.94, XVI.51), virulent to *Pc50* and avirulent to *Pc50-5*, were used for allelism tests and segregation analysis to confirm the distinctiveness and determine monogenic inheritance of *Pc50-5* in the host-pathogen experiment. Phenotype segregation consistent with the ratio of monogenic inheritance was observed within the F_2_ generation of Pc50 × Pc50-5, Kasztan × Pc50-5 and Bingo × Pc50-5 populations (*p*-value > 0.326). In the Pc50 × Pc50-5 population, the F_2_ progeny segregated as follows: 70 resistant (R):19 susceptible (S) for both *P. coronata* races (χ2 = 0.54; *p*-value = 0.462), confirming the distinctiveness of *Pc50-5* from *Pc50* and excluding their allelicity. The detailed results for the Kasztan × Pc50-5 F_2_ population were: 155 R:45 S for the I.94 *P. coronata* race (χ2 = 0.67; *p*-value = 0.414) and 148 R:52 S for the XVI.51 race (χ2 = 0.1; *p*-value = 0.744). Both races used to screen 140 F_3_ families of the Kasztan × Pc50-5 F_2_ population resulted in 36 homozygous resistant:68 segregating:36 homozygous susceptible lines (χ2 = 0.11; *p*-value = 0.94). F_2_ progeny of Bingo × Pc50-5 segregated into 70 R:20 S in case of the I.94 race (χ2 = 0.24; *p*-value = 0.62), and 64 R:26 S for the XVI.51 race (χ2 = 0.962; *p*-value = 0.326). Bingo × Pc50-5 F_3_ families segregated into 15 homozygous resistant:29 segregating:10 homozygous susceptible lines (χ2 = 0.103; *p*-value = 0.59) for both crown rust races (Table 2). Phenotype segregation within both populations, Kasztan × Pc50-5 and Bingo × Pc50-5, confirmed the monogenic pattern of inheritance.

### 2.2. Identification of DArTseq and SilicoDArT Markers Correlated with Pc50-5 Segregation Pattern and SCAR Marker Design

DArT*seq* genotyping of 45 previously phenotyped F_2_ plants of Kasztan × Pc50-5 yielded 34,100 codominant DArT*seq* and 52,301 dominant silicoDArT sequences, 22 of which showed a highly correlated segregation with the segregation pattern of *Pc50-5* dominant and recessive alleles. For DArT*seq* and silicoDArT sequences meeting the criteria for PCR primers design, SCAR (sequence-characterized amplified region) primer pairs were synthesized, but only a few resulted in the properly segregating PCR products (Table 3). Furthermore, 5426978_SCAR and 24031809_SCAR, converted from sequencing markers to PCR markers, co-segregated with the dominant *Pc50-5* allele. Surprisingly, 58163643_SCAR amplified two products, one 69 bp long, co-segregating with the dominant *Pc50-5* allele (58163643_1), and the other, 675 bp long, co-segregating with the recessive *pc50-5* allele. The PCR product obtained for homozygous susceptible genotypes (58163643_2) was subjected to Sanger sequencing (Table 3), which demonstrated that the 619 bp long internal fragment was replaced by only 10 nucleotides in resistant forms.

### 2.3. Linkage Analysis, Chromosomal Assignment and Marker Validation

**Figure 2:**
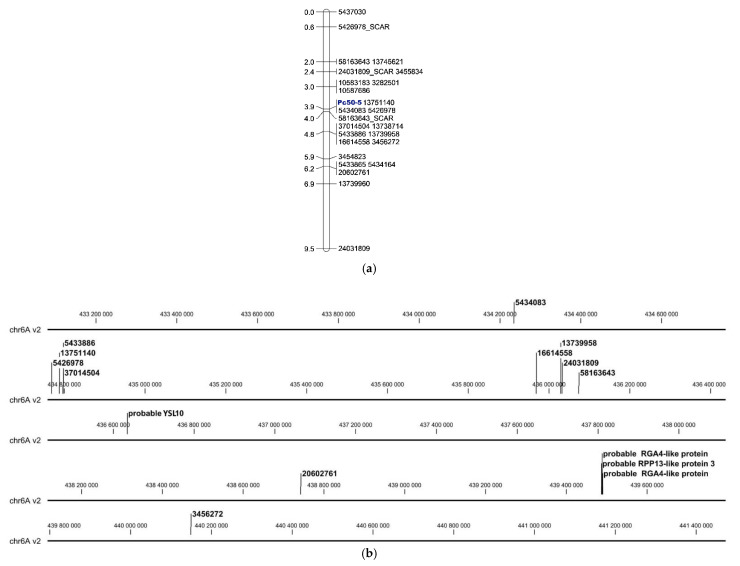
Partial linkage map of markers for *Pc50-5* gene (**a**) and its graphical assignment to chromosome 6A of the current version of the oat reference genome (*Avena sativa*-OT3098 v2, PepsiCo) [27] (**b**).

Twenty-two selected DArT*seq* and silicoDArT markers linked to the *Pc50-5* gene were used to form a partial linkage group (Figure 1). PCR reactions were performed using previously crown rust phenotyped and DArT*seq*-genotyped DNA of Kasztan x Pc50-5 F_2_ plants with three converted SCAR markers. These markers were mapped from 0.6 cM (5426978_SCAR) through 2.4 cM (24031809_SCAR) to 4.0 cM (58163643_SCAR) on the partial linkage group (Figure 2a).

The BLASTN analysis performed using marker sequences linked to *Pc50-5*, as a query against recently released hexaploid oat reference genome v2, identified regions located on chromosomes 5D and 4C, however most of the markers were found on chromosome 6A between 434,234,214 and 440,149,046 bp and spanned approximately 5.91 Mb (Figure 2b).

**Figure 3:**
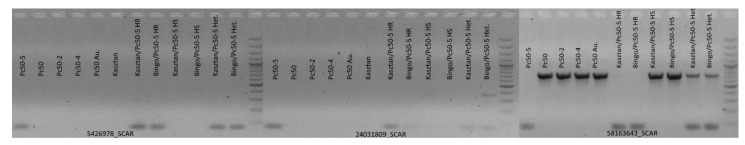
PCR markers validation on Pc50 isolines (Pendek × CW 486-l) and progeny of F_2_ Kasztan Pc50-5 and Bingo × Pc50-5 populations. HR—homozygous resistant; HS—homozygous susceptible; Het.—heterozygous.

PCR markers 5426978_SCAR, 24031809_SCAR and 58163643_SCAR used with the DNA of five *Avena* isolines developed from the Pendek × CW 486-l cross (Pc50, Pc50Au, Pc50-2, Pc50-4, Pc50-5) allowed distinguishing of Pc50-5 from other genotypes with the same pedigree (Table 4, Figure 3).

### 2.4. Sequence Homology Analysis

The three sequences identified within the region of chromosome 6A between 434,234,214 and 440,149,046 bp, where *Pc50-5* was mapped, showed high homology to coding sequences of putative disease resistance proteins. A sequence homologous to the probable metal-nicotianamine transporter YSL10 was found between 436,634,028 and 436,635,135 bp (XM_040401168.1, XM_037619029.1, XM_025969683.1). Significant similarity to the probable RGA4-like protein was found in the following regions: 439,486,403–439,487,430 and 439,489,096–439,491,139 bp (AK368786.1, XM_020344087.2, XM_037601170.1). A sequence homologous to the probable RPP13-like protein 3 was annotated between 439,486,403 and 439,487,430 bp (XM_014896855.2, XM_020344086.2, AK369251.1) (Table 5, Figure 2b).

## 3. Discussion

The donor of the *Pc50* crown rust resistance gene, *A. sterilis* CW 486-1, was collected by the Canadian-Welsh expedition in Tunisia in 1964 and crossed with *A. sativa* cultivar Pendek [23]. Šebesta and Harder [24] tested the virulence of 12 *Pc* oat differentials, including Pc50, in their study on crown rust incidence in Europe in the years 1977–1980. The latter authors observed segregation within the Pc50 line and reselected Pc50-2 and Pc50-4. Genetic analysis based on crosses indicated that each of the reselected lines contained a different major resistance gene [25].

Pc50 was also included in the reference oat line set in our previous study focused on monitoring the occurrence and harmfulness of *P. coronata* populations in Poland in the years 2013–2019 [19,20]. The study used over 600 crown rust isolates to examine the effectiveness of *Pc* resistance genes. *Pc50* was highly effective, and only a few pathotypes overcame its resistance. However, segregation of resistant and susceptible plants was found in some *P. coronata* races, and the Pc50-5 pure line, which demonstrated a very high level of crown rust resistance, was reselected. The results of crown rust reaction comparison proved a distinct infection profile of Pc-50-5 from the four other *Avena* isolines developed from the Pendek × CW 486-l cross. In addition to Pc50 derived from the Canadian seed stock of Pc differentials, Pc-50-2 and Pc50-4 from the Czech Republic (original set of Sebesta Pc differentials) as well as the Pc50 line obtained from the University of Sydney in Australia (Pc50Au) were included in the comparison. The Pc50 line presented a profile corresponding to Pc50 Au, indicating that crown rust resistance in these lines was conferred by the same gene. A distinct pattern of Pc50-5 infection allowed us to postulate the presence of a novel *Pc* gene in this line. To assess the genetic background of newly identified resistance, the Pc50-5 line was crossed with Pc50 carrier and two crown rust-susceptible Polish oat cultivars, Bingo and Kasztan. Allelism test confirmed that the resistance of Pc50 and Pc50-5 lines was conditioned by different loci. The consistent 1:2:1 ratio of resistant, segregating, and susceptible progeny proved monogenic inheritance of resistance. This makes the newly discovered gene relatively easy to transfer and incorporate into oat breeding programs, especially as other *Pc* genes are gradually being overcome by new crown rust races [28].

Breeding based on the pyramidization of *Pc* genes with different (strong or weak) effects is one of the most promising strategies for increasing the persistence of disease resistance in oat [29]. Effective identification of component genes is an essential step in successful gene pyramiding, and involves molecular marker analyses [30]. DNA markers enable fast and reliable gene identification at the early stages of plant development. In this study, DArT*seq* technology, combining conventional complexity reducing DArT system with a technique based on next-generation Illumina short-read sequencing, was used to develop markers for *Pc50-5*. This method is one of the GBS (genotyping by sequencing) variants that allows for simultaneous detection of tens of thousands of sequence-tagged markers, distributed in low-copy genomic regions [31]. Targeting sequences that are polymorphic in individuals with opposite phenotypes in the mapping population enables marker selection for a given trait and their subsequent conversion to PCR markers. The PCR-based assay is a very simple procedure; therefore, we have focused in this study on the development of specific PCR markers linked to *Pc50-5* useful for marker-assisted selection (MAS). Successful conversion of DART*seq* to PCR markers in oat was carried out, e.g., in the case of the crown rust *Pc39* gene [32]. In this study, three tightly linked SCAR markers were developed, two of which (5426978_SCAR and 24031809_SCAR) were dominant, co-segregating with the *Pc50-5* resistance allele, and located on the partial linkage group at 0.6 and 4.0 cM, respectively. The co-dominant 58163643_SCAR marker showed the best diagnostic capacity and was located the closest to *Pc50-5* at 0.1 cM. Each of the markers used with the DNA of five *Avena* isolines developed from the Pendek × CW 486-l cross (Pc50, Pc50Au, Pc50-2, Pc50-4, Pc50-5) allowed distinguishing of Pc50-5 from other genotypes with the same pedigree. Such easy-to-use PCR-based markers can facilitate the utilization of *Pc50-5* in crown rust resistance breeding programs.

*A. sativa* is a complex object of genetic research due to the large size of the allohexaploid genome with the constitution of AACCDD and basic chromosome number of x = 7 [33]. Numerous chromosomal rearrangements, a high proportion of repetitive elements as well as multigene families make it very difficult to determine gene locations and develop linkage maps [34,35,36]. The increasing availability of high-throughput sequencing methods as well as the commitment of the oat community involved in the project led to the development of a complete high-quality reference genome sequence of the hexaploid oat line OT3098 [27]. Release v2 was constructed by using short and long-read DNA sequencing technologies and improved hifiasm assembler. This allowed us to define the putative location of *Pc50-5* on chromosome 6A based on the homology of the identified markers to the reference genome.

Three sequences were identified within the marked genome region on the proximal part of chromosome 6A encoding putative disease resistance proteins homologous to probable metal-nicotianamine transporter YSL10, probable RGA4-like protein and probable RPP13-like protein. YSLs (yellow stripe-like) belong to the oligopeptide transporter family, one of the major groups of membrane-bound integral proteins that are involved in the transfer, detoxification or remobilization of metals [37]. YSL metal transporters, which are complexed with nicotianamine, are involved in metal-phytosiderophores transport and pathogen-induced defense through the regulation of salicylic acid (SA)-induced signaling [38]. Salicylic acid, in turn, has been shown to be a crucial modulator of plant immunity [39].

RPP13 (recognition of *Peronospora parasitica* 13) was first identified in *Arabidopsis thaliana* resistant to downy mildew. This is a typical R gene coding for a protein with a nucleotide-binding site (NBS), a series of leucine-rich repeats (LRR) at the C terminus and a coiled-coil domain (CC) at the N terminus [40]. RPP13 triggers disease resistance by recognizing the effector ATR13 [41]. The homologues of the RPP13 gene identified in other plant studies were found to play a key role in resistance to fungi, bacteria and viruses. RPP13-like genes differ in the position and peptide length of the LRR domains and participate in resistance of tomato to yellow leaf curl virus [42], grape powdery mildew [43], sugarcane leaf shedding [44], wheat powdery mildew [45] and barley powdery mildew [46].

RGA4 is a gene from rice (*Oryza sativa* L.) encoding NB-LRR protein, which in combination with RGA5, is required to confer resistance against *Magnaporthe oryzae* [47]. Most of the cloned rice blast resistance genes encode CC-NB-LRR proteins, and in several cases, these genes function in pairs rather than as individual NB-LRR proteins [48]. RGA5 acts as an Avr receptor and RGA4 is involved in the activation of resistance signaling [49].

Despite the occurrence of virulence to Pc50-5, detected in recent years in surveys, its frequency was very low, indicating that it may be beneficial to use Pc50-5 in oat breeding for crown rust resistance. An additional advantage is the availability of easy-to-use PCR-based markers allowing for tracking of gene allele flow in hybrids. Future research may focus on the functional characterization of candidate genes and marker development based on causative mutations for molecular breeding programs.

## 4. Materials and Methods

### 4.1. Plant Material

The main object of the study was *Pc50-5*, a race-specific seedling crown rust resistance gene, effective at all growth stages, selected from the differential Pc50 line with the *Avena sterilis* L. CW 486-1 × Pendek pedigree. Crown rust reaction comparison and PCR markers validation was performed on Pc50 isolines obtained from the Cereal Research Centre AAFC, Winnipeg, Canada (Pc50), and The University of Sydney, Plant Breeding Institute, Cobbitty, Australia (Pc50Au.), as well as Pc50-2 and Pc50-4 from the Crop Research Institute, Prague-Ruzyně, Czech Republic. The distinctiveness of the crown rust resistance gene *Pc50-5* from *Pc50* was examined on 89 F_2_ plants of the population derived from the crossing of Pc50 × Pc50-5 lines in the allelism test. The mode of *Pc50-5* gene inheritance was studied in two F_2_ and F_3_ populations resulting from crosses between the Pc50-5 line and two crown rust susceptible Polish oat cultivars, Bingo and Kasztan [50]. In the Kasztan × Pc50-5 and Bingo × Pc50-5 populations, 200 and 90 F_2_ plants and 140 and 54 F_3_ families were analyzed, respectively. Parental forms and 45 F_2_ plants of Kasztan × Pc50-5 were also used for genotyping.

### 4.2. Crown Rust Inoculation and Disease Rating

*P. coronata* f. sp. *avenae* pathotypes used in the study were selected from a wide collection of single-pustule isolates derived from populations collected in Poland in the years 2010–2019, dried and stored in 1.5-mL microfuge tubes at −70°C [19,20,51]. Before inoculation, crown rust urediniospores were heat-shocked for 4 min at 42 °C and multiplied on leaf fragments of the susceptible oat cultivar Kasztan [50] using the host-pathogen method of Hsam et al. [52], originally used for *Blumeria graminis* f. sp. *avenae* and modified by Paczos-Grzęda and Sowa [19]. Tests were conducted on the first leaves of 10-day-old seedlings. One leaf from each seedling was cut into two 3-cm-long fragments, which were divided into separate culture plates with agar (0.6%) and benzimidazole (3.4 mM). Inoculation was performed in a settling tower by spreading urediniospores on plant material at a density of 500–700 spores/cm^2^. Plates were incubated for 10–12 days in a growth chamber at 18 °C with 70% humidity, light intensity of approximately 4 kLx under a 16-h photoperiod. Infection type (IT) data were assessed on a modified 0–4 qualitative scale as follows: 4—susceptible, large to moderately large pustules with little or no chlorosis; 3—moderately susceptible, moderately large pustules surrounded by extensive chlorosis; 2—moderately resistant, small pustules surrounded by chlorosis; 1—resistant, chlorotic or necrotic flecking; and 0—highly resistant, no visible reaction [53,54]. If disease symptoms were scored as 4 or 3, the genotype was classified as susceptible and the rest as resistant.

After the seedling tests, all individuals were planted in the experimental farm of the University of Life Sciences in Lublin (Czesławice 51°18′ N, 22°15′ E). F_2:3_ generation seeds were collected and grown in plug trays filled with a universal substrate containing peat.

Five *Avena* isolines developed from the Pendek × CW 486-l cross (Pc50, Pc50Au, Pc50-2, Pc50-4, Pc50-5) were tested with 14 *P. coronata* pathotypes (Table 6) as described above. The resistance response was coded as 0, while susceptibility as 1. Every line was represented by 10 plants.

For allelism, two *P. coronata* pathotypes, i.e., I.94 and XVI.51 (Table 6), were used on 89 F_2_ plants of the Pc50 × Pc50-5 population in the host-pathogen tests, conducted as described above, except that one leaf from each seedling was cut into two 3-cm-long fragments.

The same two *P. coronata* races were used to target the segregation of the *Pc50-5* gene. F_2_ lines of Kasztan × Pc50-5 and 54 F_2_ lines of Bingo × Pc50-5 (142 lines in total) were phenotyped using inoculation and assessment techniques consistent with the methods of Sowa et al. [32]. Approximately 16 F_3_ plants from each line were tested.

### 4.3. DNA Extraction and Genotyping

Total genomic DNA was extracted from mechanically disrupted tissue of leaf material of F_2_ individuals and parental forms using the DNeasy Plant Mini Kit (Qiagen). DNA integrity and quality were evaluated by electrophoresis on a 1% agarose gel. DNA concentration was determined using a NanoDrop2000 spectrophotometer and normalized to 100 ng·μL^−1^.

To identify *Pc50-5*-linked markers, genotyping of 47 F_2_ plants, representing the Kasztan × Pc50-5 population, and sequencing of parental forms using DArT*seq*, was performed at Diversity Arrays Technology Pty Ltd. (DArT P/L, Canberra, Australia). DArT*seq* technology, combine the DArT technique with next-generation sequencing, as described by Courtois et al. [55]. DNA libraries were generated using genomic complexity reduction technology [56] by digestion of DNA samples with PstI and TaqI restriction enzymes (NEB) and ligation with corresponding adaptors. Only PstI-TaqI fragments were effectively PCR amplified followed by sequencing on Illumina Hiseq 2500. Sequencing data were processed using proprietary DArT analytical pipelines providing two types of markers: silicoDArT presence/absence variants (PAVs) analogous to microarray DArTs, but extracted in silico from sequences obtained from genomic representations and DArT*seq* single-nucleotide polymorphisms (SNPs) in fragments present in the representation. Both sequence types were BLASTed against a reference genome sequence *Avena sativa* OT3098 v2, PepsiCo [27], with an expected value (E) < 10 ^−10^ and minimum base identity >95% as blast criteria.

### 4.4. PCR Primer Design and SCAR Marker Validation

SilicoDArT and DArT*seq* markers with segregation pattern closest to the crown rust resistance phenotype in the study population and a sequence length of not less than 50 nucleotides were selected for SCAR (sequence-characterized amplified region) marker development. DNA sequences were analyzed with the BioEdit sequence alignment editor v. 7.0.5.3 [57] and used to design PCR primers with NCBI primer blast [58] and Primer3 software with the default options [59].

PCR was carried out in 10-μL reactions containing 20 ng of template DNA, 1 × JumpStart Taq ReadyMix (Merck) and 0.35 µM of each forward and reverse oligonucleotide primer. The PCR thermal profile consisted of an initial hold at 94 °C for 4 min, followed by 38 cycles of 94 °C for 30 s, annealing temperature for 45 s and 72 °C for 1 min, followed by a final elongation at 72 °C for 7 min. Amplification products were separated on a 1.5% agarose gel containing 5 μg/mL EtBr in 1xTBE buffer (90 mM Tris-borate, 2 mM EDTA, pH 8.0). Gene Ruler 100 bp Plus DNA Ladder was used to determine molecular weight of the products. Fragments were visualized under UV transilluminator and photographed.

PCR products of unexpected length were sequenced using the Sanger method at Genomed (Warsaw, Poland).

### 4.5. Statistical and Linkage Analyses

Infection severity scores of isolines (Pc50, Pc50Au, Pc50-2, Pc50-4, Pc50-5) transformed into a 0/1 matrix (0—resistant, 1—susceptible) were used to conduct clustering and construct a dissimilarity dendrogram based on the unweighted pair-group method (UPGMA) in the PAST 3.19 software [60]. Groups were determined using the Dice coefficient [61] with 1000 bootstraps.

Chi-squared (χ2) analyses of the crown rust infection type (IT) data from the F_2_ and F_3_ progeny were tested for goodness-of-fit of the observed to expected segregation ratios.

MapDisto 2.0 software [62] was used to create a partial linkage group from DNA marker data based on a minimum LOD (logarithm of odds) threshold score of 3.0 and a maximum recombination fraction threshold of 0.3. Individuals with >20% of missing data were omitted. Marker order was determined by the Seriation II method based on the Seriation algorithm [63] with the use of the SARF (Sum of Adjacent Recombination Frequencies) criterion [64].

### 4.6. Sequence Data Analysis

Marker sequences linked to *Pc50-5* were used to flank the region of the current version of the oat reference genome (*Avena sativa* OT3098 v2, PepsiCo) [27]. The region was analyzed, and the obtained transcripts were filtered based on their physical positions to exclude redundancy; they were subsequently used to search for orthologous sequences with BLASTn at http://www.ncbi.nlm.nih.gov (accessed on 16 July 2021) of the National Center for Biotechnology Information (NCBI) using the Nucleotide Collection Database [65]. The threshold parameter was established at 10^−7^, with E-value hits below this cutoff point considered significant.

## Figures and Tables

**Table 1:** Infection profiles of oat isolines derived from Pendek × CW 486-l crossing based on the reaction to *P. coronata* race infection.

	*Puccinia Coronata* Race *													
	I.94	XVI.51	I	3.2	13.1	37.58 K	94.1/4	I.94 (63)	230	233	241	241/19	254	257
Pc50	1	1	0	0	0	0	0	0	0	0	0	0	0	0
Pc50 Au	1	1	0	0	0	0	0	0	0	0	0	0	0	0
Pc50-5	0	0	1	0	0	0	0	0	1	1	0	1	1	1
Pc50-2 Cz	1	1	1	1	1	0	1	1	1	1	0	1	1	1
Pc50-4 Cz	1	1	1	1	1	1	0	1	1	1	0	1	1	1

* Resistance phenotype: 1—susceptible, 0—resistant.

**Table 2:** Segregation ratios of F_2_ progeny and F_3_ families of populations Pc50/Pc50-5, Kasztan/Pc50-5 and Bingo/Pc50-5 when inoculated with *P. coronata* races I.94 and XVI.51.

Population	Generation	*Puccinia Coronata* Race	Resistant	Segregating	Susceptible	Ratio	Χ^2^	*p*-Value
Pc50/Pc50-5	F_2_	I.94	70	-	19	3:1	0.54	0.46
	F_2_	XVI.51	70	-	19	3:1	0.54	0.46
Kasztan/Pc50-5	F_2_	I.94	155	-	45	3:1	0.67	0.41
	F_3_	I.94	36	68	36	1:2:1	0.11	0.94
	F_2_	XVI.51	148	-	52	3:1	0.1	0.74
	F_3_	XVI.51	36	68	36	1:2:1	0.11	0.94
Bingo/Pc50-5	F_2_	I.94	70	-	20	3:1	0.53	0.47
	F_3_	I.94	15	29	10	1:2:1	1.03	0.59
	F_2_	XVI.51	64	-	26	3:1	0.53	0.47
	F_3_	XVI.51	15	29	10	1:2:1	1.03	0.59

**Table 3:** DNA sequences used for SCAR markers design.

Sequence Name	Sequence (5′–3′)	Primers Annealing Temp. (°C)
5426978	**TGCAGGTATATCCTCTCCG**AAGGAGTCGCTCAACGCC**ACGACAGTGGAGGAGAATTC**	67
58163643_1	**TGCAGCCTACAGGCAAGTG**GTGGAGTGGATAC*TACAGCCCAC*AGG**GAACGCTACTCTGGACATCGA**ATA	62
58163643_2	**TGCAGCCTACAGGCAAGTG**GTGGAGTGGATAC*GATCGATACGACGGACCATGAATCCATGAGGATCCCTGCAAGTTGGGTCGCGGCCGTGCGTGAGAGGAGATCCTCCTCGGAAAGACGTGAACGGATCACGGACAGGAAGCATTCTATCGCGGCAGCATGCATGCCACGCTTTTCTTTCTCTTCACCGAATCAACAAAAAGCACAGGGGAAAGTATACTGCTTTGGTGAAGTGGGATCGAATCGTATCCCGTGGCACGAGCTGACGAGCGATTAGATCAATCCCAAATGCATGGAAAGCGACCGGGATTATTAGGGATATTATAAGAGCAAGAACGATAGTATAGCTAGCAGGTGGCTATATGATGCCGTAATGTTAACTTGCACAGTAGGGTTGGCTATAAGATTGACTATTAGATTAGTGTTATCTTCTCTCTTTCTTTTTCCTCTCGTTTAATGTTTTTTGTCTAGGAGCAAGTGTAGAGCTGACTCTTGCATGAGAGCCAGCAACTCGTAGTTTTGTTTATCTCTCTCTTTTACATAGACAAAAATGCCTAATCAGCAGGCTTATAGTCCACTATTGTACTTGCTCTAACTGGAACAAGAGGGACCACCGCTTGCTTTCAGTGCCACAATGGGCCAAATTGTCCCT*AGG**GAACGCTACTCTGGACATCGA**	
24031809	TG**CAGCTGCTGATTAACAAAAGCTCAC**ATTAGTAAAGCCGAAGCCT**CTCCGCGCACTCACGGC**AAAGTC	66

The bold, underlined regions are the sequences used for SCAR primer design. The italics mark sequences differing resistant and susceptible alleles of 58163643_SCAR.

**Table 4:** PCR markers validation on Pc50 isolines (Pendek × CW 486-l).

Oat Line	5426978_SCAR	58163643_SCAR	24031809_SCAR
Pc50	-	B	-
Pc50Au	-	B	-
Pc50-2	-	B	-
Pc50-4	-	B	-
Pc50-5	A	A	A

A—*Pc50-5* resistant allele carrier; B—*pc50-5* susceptible allele carrier.

**Table 5:** Detailed information on predicted genes within the region where *Pc50-5* was mapped to oat reference genome (*Avena sativa*-OT3098 v2, PepsiCo) [27].

Physical Position (bp) on Chromosome 6A	Name	Homologous Accessions
436634028–436635135	probable metal-nicotianamine transporter YSL10	*Aegilops tauschii* subsp. *strangulata* XM_040401168.1 *Triticum dicoccoides* XM_037619029.1 *Panicum hallii* XM_025969683.1
439486403–439487430 439489096–439491139	probable RGA4-like protein	*Hordeum vulgare* subsp. *vulgare* AK368786.1 *Aegilops tauschii* subsp. *strangulata* XM_020344087.2 *Triticum dicoccoides* XM_037601170.1
439486403–439487430	probable RPP13-like protein 3	*Brachypodium distachyon* XM_014896855.2 *Aegilops tauschii* subsp. *strangulata* XM_020344086.2 *Hordeum vulgare* subsp. *vulgare* AK369251.1

**Table 6:** Virulence spectrum of *P. coronata* f. sp. *avenae* pathotypes used to differentiate Pc50 isolines and target segregation ratio in the Kasztan × Pc50-5, Bingo × Pc50-5 and Pc50 × Pc50-5 progeny.

Race No.	Phenotype Code ^1^	Virulence to Supplemental Differentials
I.94	TBLN	Pc14, Pc35, Pc57, Pc96, Pc97, Pc98, Pc103-1
	TBLN	Pc35, Pc57, Pc96, Pc97, Pc98, Pc103-1
XVI.51		Pc14, Pc35, Pc57, Pc96, Pc97, Pc98, Pc103-1
I	NJBM	Pc36, Pc57, Pc61, Pc67, Pc70, Pc71, Pc94, Pc96, Pc98, Pc103-1
3.2	SBBL	Pc14, Pc35, Pc55, Pc67, Pc96, Pc97, Pc98, Pc103-1
13.1	BLBG	Pc55, Pc98, Pc103-1
37.58K	BLBB	Pc36, Pc63
94.1/4	JBLL	Pc35, Pc57, Pc96, Pc97, Pc98, Pc103-1, Pc104
I.94(63)2018	LDQB	Pc14, Pc35, Pc36, Pc57, Pc104
230	LQBC	Pc35, Pc36, Pc60, Pc61, Pc63, Pc70, Pc91
233	NGBB	Pc36, Pc61, Pc70, Pc71, Pc94, Pc103-1
241	LDRB	Pc14, Pc35, Pc36, Pc57, Pc67, Pc103-1, Pc104
241/19	NSGC	Pc35, Pc61, Pc63, Pc71, Pc97
254	BRCH	Pc35, Pc55, Pc57, Pc61, Pc63, Pc67, Pc71, Pc104
257	BRMH	Pc55, Pc61, Pc63, Pc67, Pc70, Pc71, Pc94, Pc98

^1^ Phenotype code based on the standard differentials set.
